# The Efficacy of Botulinum Toxin Type A (BTA) in the Treatment of Hypertrophic Scars and Keloids: A Systematic Review and Meta-Analysis of Randomized Controlled Trials

**DOI:** 10.7759/cureus.71161

**Published:** 2024-10-09

**Authors:** Elshymaa E Raslan, Basel H Bakhamees, Tafe A Howsawi, Layan S Alshmrani, Araa G Alruwaili, Abdulrahman Y Alhashmi, Shatha M Aldor, Wiam M Alhoshani, Maryam Y Almuslem, Rana A Alharbi, Afaf H Homeirani, Sarah K Alkhorayef, Mohammed A Alqahtani

**Affiliations:** 1 Surgery, King Khalid Hospital, Tabuk, SAU; 2 Faculty of Medicine, King Abdulaziz University, Jeddah, SAU; 3 Emergency Medical Service, King Saud Bin Abdulaziz University for Health Sciences, Jeddah, SAU; 4 College of Medicine, King Khalid University, Abha, SAU; 5 Surgery, Al Jouf University, Sakakah, SAU; 6 Surgery, King Abdulaziz University, Jeddah, SAU; 7 College of Medicine, Ibn Sina National College of Medicine, Jeddah, SAU; 8 College of Medicine, Qassim University, Ar Rass, SAU; 9 Facility of Medicine, King Faisal University, Al-Ahsa, SAU; 10 College of Medicine, Princess Nourah Bint Abdulrahman University, Riyadh, SAU; 11 College of Medicine, University of Tabuk, Tabuk, SAU; 12 Medicine and Surgery, Ibn Sina National College of Medicine, Jeddah, SAU; 13 Plastic and Reconstructive Surgery, University Medical Center Groningen, Groningen, NLD

**Keywords:** botulinum toxin type a, bta, hypertrophic, meta-analysis, scars

## Abstract

Hypertrophic scars cause significant physical and emotional discomfort. Botulinum toxin type A (BTA) has shown promising outcomes in reducing scar formation. Research suggested its effectiveness in managing hypertrophic scars and keloids. This systematic review and meta-analysis were reported according to the Preferred Reporting Items for Systematic Reviews and Meta-Analyses (PRISMA) guidelines, including randomized controlled trials (RCTs). The included studies involved patients with hypertrophic scars treated with BTA. Five databases were searched from inception to August 2024. Studies were screened and selected by two independent reviewers. Data on study design, patient demographics, and interventions was extracted. The risk of bias (ROB) was assessed using the ROB revised tool developed by Cochrane (ROB2). Meta-analysis was performed using RevMan Web (The Cochrane Collaboration, London, UK) with a random-effects model due to high heterogeneity, calculating mean differences for the primary and secondary outcomes. Outcomes included Vancouver Scar Scale (VSS) scores, scar thickness, vascularity, pliability, and pigmentation. The systematic review identified 182 records from five databases. The screening process resulted in seven studies included in the final analysis after assessment for eligibility. The efficacy of BTA in treating hypertrophic scars and keloids was assessed. The meta-analysis showed that BTA significantly improved VSS scores, with a pooled mean difference of -1.69 (P = 0.01). However, BTA did not show a statistically significant effect on reducing scar height/thickness or improving vascularity. Scar pliability was significantly improved by BTA, with a pooled mean difference of -0.76 (P = 0.002), while there was no significant effect on pigmentation. High heterogeneity was observed across the studies. BTA could be used as an effective treatment for the components of hypertrophic scars and keloids, especially regarding their pliability and improving scar quality. However, for functions such as the change in scar height, vascularity, and pigmentation, more research is still required among larger RCTs. Future research should focus on refining the treatment regimens and the mode of action of BTA to the scar tissue.

## Introduction and background

Keloids and hypertrophic scars are both forms of excessive scar tissue, but they differ in key aspects. Keloids grow beyond the boundaries of the original wound, often becoming larger and firmer, and can continue expanding over time. They may cause itching or discomfort and are more common in people with darker skin tones or a genetic predisposition. In contrast, hypertrophic scars remain confined to the original wound area, tend to be less raised, and may improve over time without spreading. While keloids often resist treatment and have a high recurrence rate, hypertrophic scars generally respond better to therapies and are less likely to recur [1]. These conditions are regarded as a burden both for the patient and the clinician, as they cause physical, emotional, and psychological discomfort [2].

The incidence and prevalence of hypertrophic scars and keloids vary, with keloids occurring equally in men and women. However, they are more common in younger individuals of African, Asian, and Hispanic descent [3]. In black and Hispanic populations, the incidence of developing keloids ranges between 4 and 16%, while in Caucasian populations, incidences are below 1% [4].

Botulinum toxin (BXT) is used in medical and esthetic treatments to immobilize muscles and their cells. A finding from one study suggests that BXT can be as effective as steroids in decreasing keloid bulk and redness and is beneficial in alleviating pain and itch. However, evidence supporting BXT's role in managing keloids following surgical removal is limited [5].

**Figure 1:**
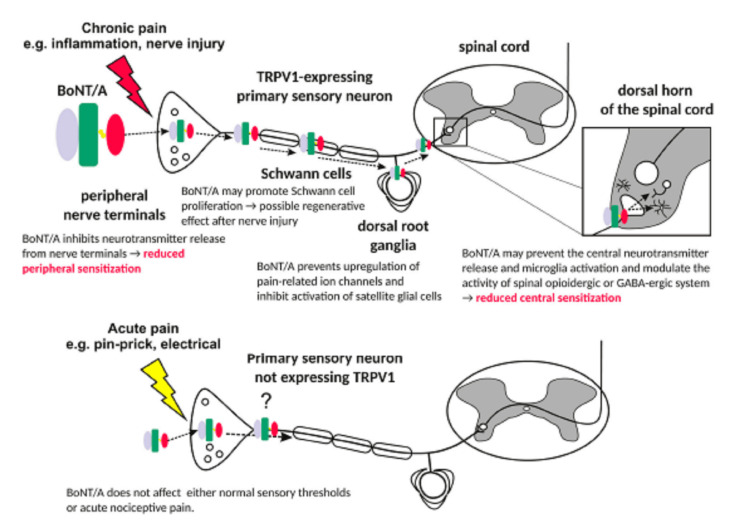
Actions of BTA along the pain pathway. BoNT/A: botulinum neurotoxin A; TRPV1: transient receptor potential vanilloid 1; GABA: gamma-aminobutyric acid. Figure is adapted from Matak et al. [7].

Botulinum toxin type A (BTA) was first developed for the management of neuromuscular disorders and has gained attention for its effective function in modulating wound healing and enhancing scar outcomes. The mechanism by which BTA works on scars involves the inhibition of neuromuscular junctions, resulting in a reduction in muscle activity and tension on the affected wound. This reduction in tension may decrease the mechanical forces that contribute to scar formation, thus resulting in more favorable cosmetic outcomes. Furthermore, BTA has been suggested to possess anti-inflammatory properties, which may contribute to its efficacy in scar management [6]. Despite the advantages BTA may offer, with new data obtained in recent years improving the understanding of its actions, more experimental and clinical studies appear to be required. Figure 1 illustrates the mechanism of action of BTA [7] properties, which may contribute to its efficacy in scar management [6].

Several studies have been developed on the effectiveness of BTA in the management of hypertrophic scars and keloids. Current research shows that most clinicians have confirmed the clinical effectiveness of BTA in the prevention and treatment of pathological scars. However, its mode of action and combination therapy need more research [8]. For instance, in 2020, Zhang et al. conducted a systematic review and meta-analysis that showed that BTA injections are effective in preventing postoperative scars and improving the aesthetic appearance of existing scars [9]. Additionally, in 2021, another meta-analysis by Qiao et al. confirmed these findings, emphasizing the role of BTA in improving scar quality and reducing hypertrophic scar formation [10].

Previous research has also compared the outcomes of BTA with other treatment modalities. For example, Naumann and Jankovic (2004) conducted a systematic review and meta-analysis to evaluate the safety profile of BTA, finding it to be well-tolerated across various indications, including scar treatment [11].

It is noteworthy to our knowledge that previous research emphasizes a more systematic examination of the efficacy of BTA on various clinical outcomes. Therefore, this systematic review and meta-analysis will evaluate the efficacy of BTA in the treatment of hypertrophic scars and keloids, providing valuable insights for clinicians and researchers.

## Review

Methods

Methodology

The study was conducted according to the principles of the Cochrane Handbook for Systematic Reviews of Interventions, version 6, and reported following the Preferred Reporting Items for Systematic Reviews and Meta-Analyses (PRISMA) guidelines [12].

Eligibility Criteria

Types of studies included: We included only randomized controlled trials (RCTs) published in English without setting limitations on the publication time.

Participants

Studies included patients who have hypertrophic scars caused either after surgery or injury and exposed to BTA injection.

Interventions

A direct comparison between BTA and placebo was conducted.

Exclusion Criteria

Conference abstracts, duplicate reports, case reports, observational studies, review articles, editorials, clinical guidelines, and single-arm studies were excluded.

Search Strategy

Online search was carried out using five databases: MEDLINE, PubMed, Cochrane Central Register of Controlled Trials (CENTRAL), Web of Science, and Scopus. We did not use any search filters, and the search encompassed the period from inception till August 2024. The terms used for searching included ("Botulinum Toxin Type A" or "BTA" or "BoNT-A") AND (("Hypertrophic scars" or "Keloids")) AND (("Scar management" or "Scar treatment")). No search filters were applied to limit the publication time, and the search was limited to studies published in English.

Selection of Studies

The processes of online search, screening the titles and abstracts, and revising the full text of relevant articles were conducted by two independent researchers. Full texts of relevant studies were reviewed for eligibility. Any disagreements were resolved by consensus.

Data Extraction

Data were extracted including study design, patient demographics, interventions, and outcomes (e.g., Vancouver Scar Scale (VSS)).

Measured Outcomes

The primary outcome was the severity of the scar, which was measured using the Vancouver Scar Scale (VSS) score. The secondary outcomes included the individual parameters of the VSS score, specifically scar thickness, pliability, vascularity, and pigmentation.

Assessment of the Risk of Bias in the Included Studies

We assessed the risk of bias (ROB) using the revised version of the Cochrane ROB tool for RCTs (ROB2) [13], as all included studies were found to be RCTs. The ROB2 tool comprises five domains: randomization, deviations from the assigned treatment, missing data, measurement of the outcome, and selective reporting of the outcomes and results. Moreover, the overall ROB was assessed by selecting the highest level of ROB out of the five domains. Data were visualized using the Robvis visualization tool [14].

Data Synthesis

The meta-analysis was conducted using the online version of Revman Web (The Cochrane Collaboration, London, UK), applying a random-effects model due to the high heterogeneity observed among the included studies [15]. Mean differences were calculated for the outcomes, including VSS score, scar height/thickness, vascularity, pliability, and pigmentation. Heterogeneity was assessed using Cochran's Q test and the I^2^ statistic. The pooled effect sizes were presented with 95% confidence intervals (CI) and p-values. The risk of bias was evaluated using the ROB2 tool.

Results

**Figure 2:**
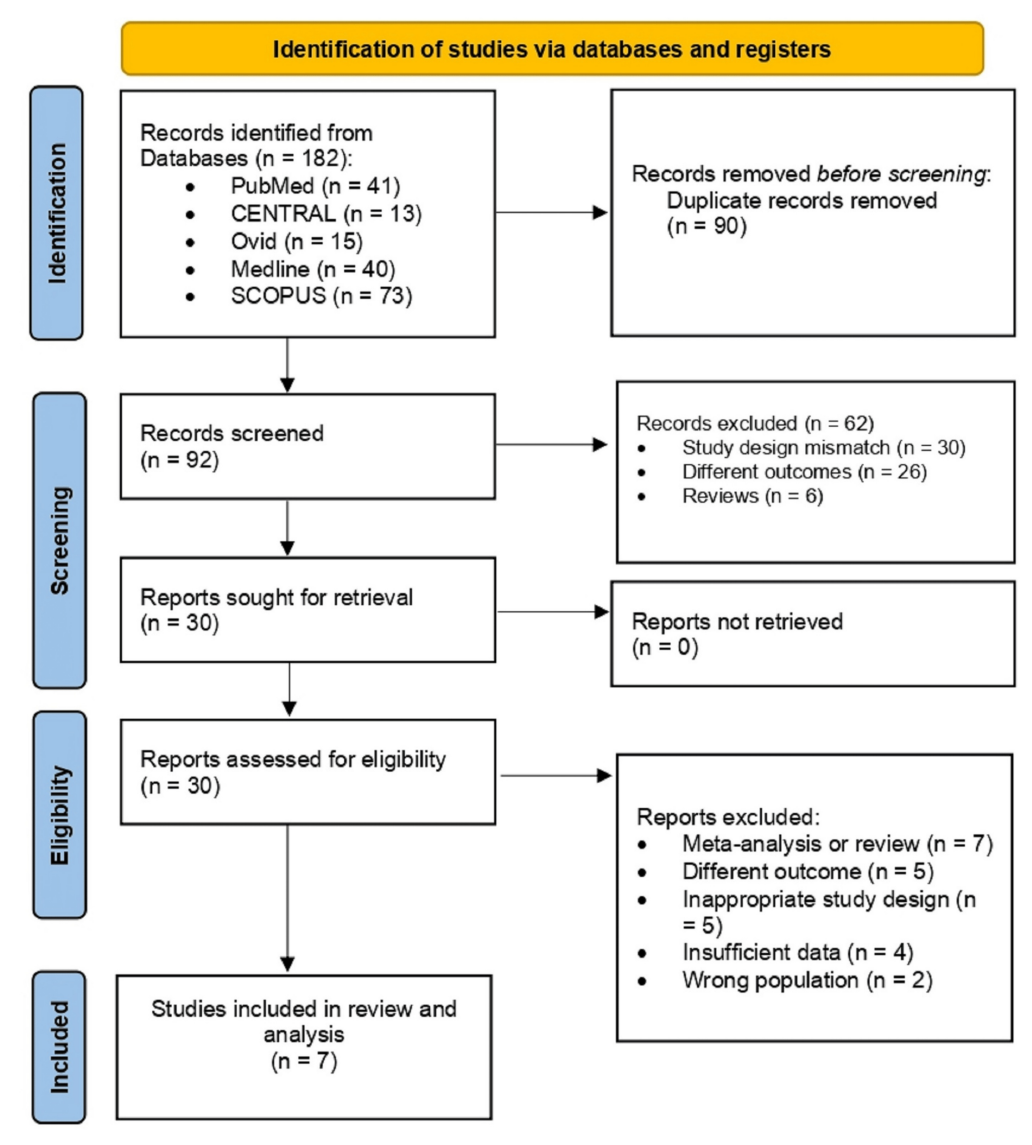
PRISMA flowchart of the screening process. PRISMA: Preferred Reporting Items for Systematic Reviews and Meta-Analyses.

The systematic review selection process started with the identification of 182 records from databases: PubMed, CENTRAL, Ovid, Medline, and SCOPUS. After removing 90 duplicates, 92 records were screened for their titles and abstracts. Of these, 62 were excluded for the following reasons: study design mismatch (n = 30), different outcomes (n = 26), or reviews (n = 6). Thirty reports were retrieved for full-text screening and were assessed for eligibility. Of these, seven meta-analyses or reviews, five inappropriate study designs, five studies with different outcomes, four with insufficient data, and two with the wrong population were excluded. In the end, seven studies were included in the final review and analysis (Figure 2) [16-22].

Characteristics of the Included Studies

Table 1 summarizes the included RCTs exploring the efficacy of BTA injections for treating hypertrophic scars and keloids. The studies were conducted across different countries, including Egypt [12,14], Iran [13,15], China [16,18], and Korea [17]. The designs of the RCTs had durations ranging from several months to years. The patient populations varied widely, including ages from two to 64 years, and the conditions treated involve post-surgical or post-burn hypertrophic scars or keloids. Each study indicated the intervention mode of administration, either intralesional or subcutaneous injections, with doses and administration schedules tailored to the specific condition and scar location. Placebo groups received either no treatment or saline injections (Table 1).

**Table 1:** Summary of the characteristics of the included studies. BTA: botulinum toxin type A; BoNT-A: botulinum neurotoxin type A; BTX-A: botulinum toxin type A; IU: international units; ml: milliliter; U: units; N: number of subjects.

Study ID	Study design	Duration of the study	N	Age (range)	Type of condition	Dose and administration	Placebo group
Tawfik et al., 2023, Egypt [16]	Randomized intra-patient comparative study.	Six months with follow-up	15	Two to 15 years	Post-burn hypertrophic scars and keloids	The lesion was injected with 5 IU/cm² every month intradermally at the periphery and into the body of the scar.	The other part of the scar/keloid is left untreated.
Abedini et al., 2022, Iran [17]	Prospective, double-blinded, split-scar, randomized placebo-controlled trial.	2018 to 2019	19	26 to 54 years	Hypertrophic scars following mammoplasty and abdominoplasty surgeries	A single treatment was performed with 5 IU/cm of BoNT-A (XEOMIN) injected intradermally into one half of the scar, Injections were administered five to 10 days postoperatively.	The placebo group received 0.9% saline injections on the opposite side of the scar.
Elshahed et al., 2020, Egypt [18]	Split-scar, double-blind randomized controlled trial	Six months	21	19 to 41 years	Hypertrophic scars	5 U/0.1 mL, with a dose adjusted to 2.5 U/cm² of the lesion. Injections were performed Intralesional once a month for a total of three months.	Intralesional injections of 0.9% saline as a placebo of the other half of the scar.
Habibi et al., 2020, Iran [19]	Double-blinded, randomized control trial	2016 to 2017, with six months follow-up	10	16 to 60 years	Keloid and hypertrophic scars	Intralesional injection at 4 U per cubic centimeter of the lesion, administered once monthly for three months.	Intralesional injections of 0.9% saline
Huang et al., 2019, China [20]	Single-center, double-blind, split-face, randomized controlled trial	11 months (2016 to 2017)	30	20 to 39 years	Hypertrophic scarring in the medial canthal area after epicanthoplasty	5 U of botulinum toxin type A in 0.1 ml of 0.9% normal saline, administered subcutaneously at two injection sites per medial canthus	0.9% Normal saline at the same dose and administration schedule as the BTA group.
Kim et al., 2019, Korea [21]	Prospective, split-scar, double-blinded, randomized controlled study	2012 to 2015 (approximately four years)	45	19 to 64 years	Hypertrophic scars in patients with forehead lacerations	5 IU/cm of BoNTA was injected into multiple intralesional and intradermal sites around the sutured area within 0.5 cm of the suture line.	Saline injections
Li et al., 2018, China [22]	Randomized, placebo-controlled, double-blind, prospective clinical trial	2016 to 2018 (approximately 15 months)	17	25 to 62 years	Hypertrophic scar development in median sternotomy wounds	0.1 mL BTA injections (five units per injection) at points 1 cm away from the wound edges, with injections placed 1 cm apart along the wound.	0.9% Normal saline at the same locations.

Meta-Analysis

**Figure 3:**
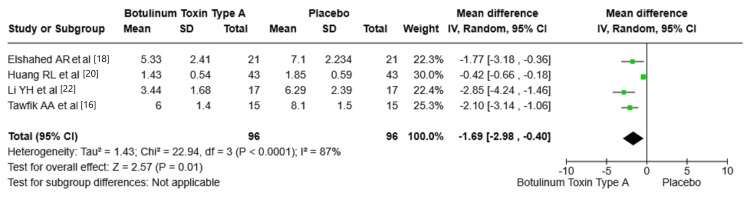
Mean difference in VSS between BTA and placebo group. VSS: Vancouver Scar Scale; BTA: botulinum toxin type A; SD: standard deviation. Source: Tawfik et al. [16], Elshahed et al. [18], Huang et al. [20], Li et al. [22].

The Vancouver Scar Scale (VSS): The VSS outcomes between botulinum toxin type A (BTA) and placebo were assessed among four studies. Elshahed et al. [18], Huang et al. [20], Li et al. [22], and Tawfik et al. [16] reported mean differences in VSS scores between the BTA and placebo groups as follows: -1.77, -0.42, -2.85, and -2.10, respectively. These mean differences indicate a consistent reduction in VSS scores with BTA. The pooled mean difference across all studies was -1.69 (95% CI: -2.98, -0.40, P = 0.01), showing a statistically significant improvement in VSS outcomes favoring BTA. The heterogeneity (I^2^ = 87%) between the included studies may be attributed to variable study designs, patient groups, or treatment approaches. Figure 3 shows the mean difference in VSS between BTA and placebo groups.

**Figure 4:**
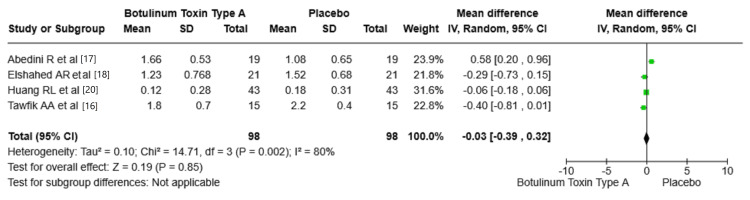
Mean difference in scar height/thickness between BTA and placebo groups. VSS: Vancouver Scar Scale; BTA: botulinum toxin type A; SD: standard deviation. Source: Tawfik et al. [16], Abedini et al. [17], Elshahed et al. [18], Huang et al. [20].

Scar height/thickness: The analysis suggests that BTA does not have a statistically significant effect on reducing scar height/thickness compared to placebo. Abedini et al. [17], Elshahed et al. [18], Huang et al. [20], and Tawfik et al. [16] reported different mean differences in scar height/thickness between the BTA and placebo groups. Abedini et al. found a mean difference of 0.58 (95% CI: 0.20, 0.96), favoring placebo. Conversely, Elshahed et al. [18] and Tawfik et al. [16] found mean differences of (-0.29 and -0.40), respectively, both favoring BTA but with no significant effect. In addition, Huang et al. [20] reported no significant difference between BTA and placebo. The pooled effect size was -0.03 (95% CI: -0.39, 0.32, P = 0.85). Figure 4 shows the mean difference in scar height/thickness between BTA and placebo groups.

**Figure 5:**
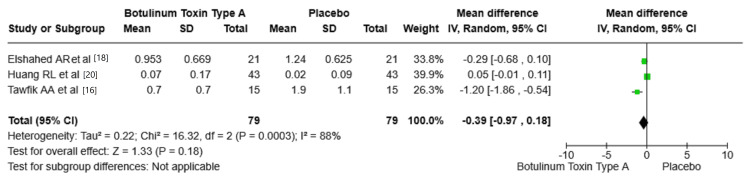
Mean difference in vascularity between BTA and placebo groups. VSS: Vancouver Scar Scale; BTA: botulinum toxin type A; SD: standard deviation. Source: Tawfik et al. [16], Elshahed et al. [18], Huang et al. [20].

Scar vascularity: The studies by Elshahed et al. [18], Huang et al. [20], and Tawfik et al. [16] provide different results regarding scar vascularity. Elshahed et al. reported a mean difference of -0.29 (95% CI: -0.68, 0.10), favoring BTA. Huang et al. found that the mean difference equaled to 0.05 (95% CI: -0.01, 0.11), slightly favoring placebo. Tawfik et al. reported a mean difference of -1.20 (95% CI: -1.86, -0.54), favoring BTA. The pooled mean difference was -0.39 (95% CI: -0.97, 0.18, P = 0.18) with no statistically significant effect of BTA compared to placebo. The heterogeneity among the studies was high (I² = 88%, P = 0.0003), indicating variable outcomes. Figure 5 shows the mean difference in vascularity between BTA and placebo groups.

**Figure 6:**
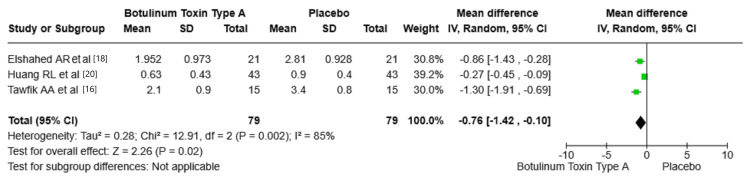
Mean difference in pliability between BTA and placebo groups. VSS: Vancouver Scar Scale; BTA: botulinum toxin type A; SD: standard deviation. Source: Tawfik et al. [16], Elshahed et al. [18], Huang et al. [20].

Scar pliability: The analysis suggests that BTA significantly improves scar pliability compared to placebo, though the effect size may vary based on the specific conditions of each study. The pooled mean difference across the three included studies was -0.76 (95% CI: -1.42, -0.10, P= 0.02), indicating a statistically significant improvement in scar pliability with BTA compared to placebo. The heterogeneity was found to be high (I² = 85%, P = 0.002). Figure 6 shows the mean difference in pliability between BTA and placebo groups.

**Figure 7:**
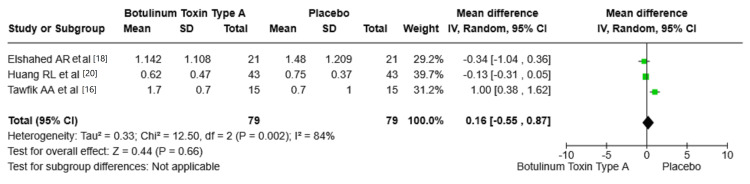
Mean difference in pigmentation between BTA and placebo groups. VSS: Vancouver Scar Scale; BTA: botulinum toxin type A; SD: standard deviation. Source: Tawfik et al. [16], Elshahed et al. [18], Huang et al. [20].

Pigmentation: The studies indicate that BTA efficacy in improving skin pigmentation did not differ from the placebo, but the results of the studies can vary, which requires further research. The pooled mean difference across all studies was 0.16 (95% CI: -0.55, 0.87, p = 0.66), with no statistically significant difference in pigmentation outcomes. Figure 7 shows the mean difference in pigmentation between BTA and placebo groups.

**Figure 8:**
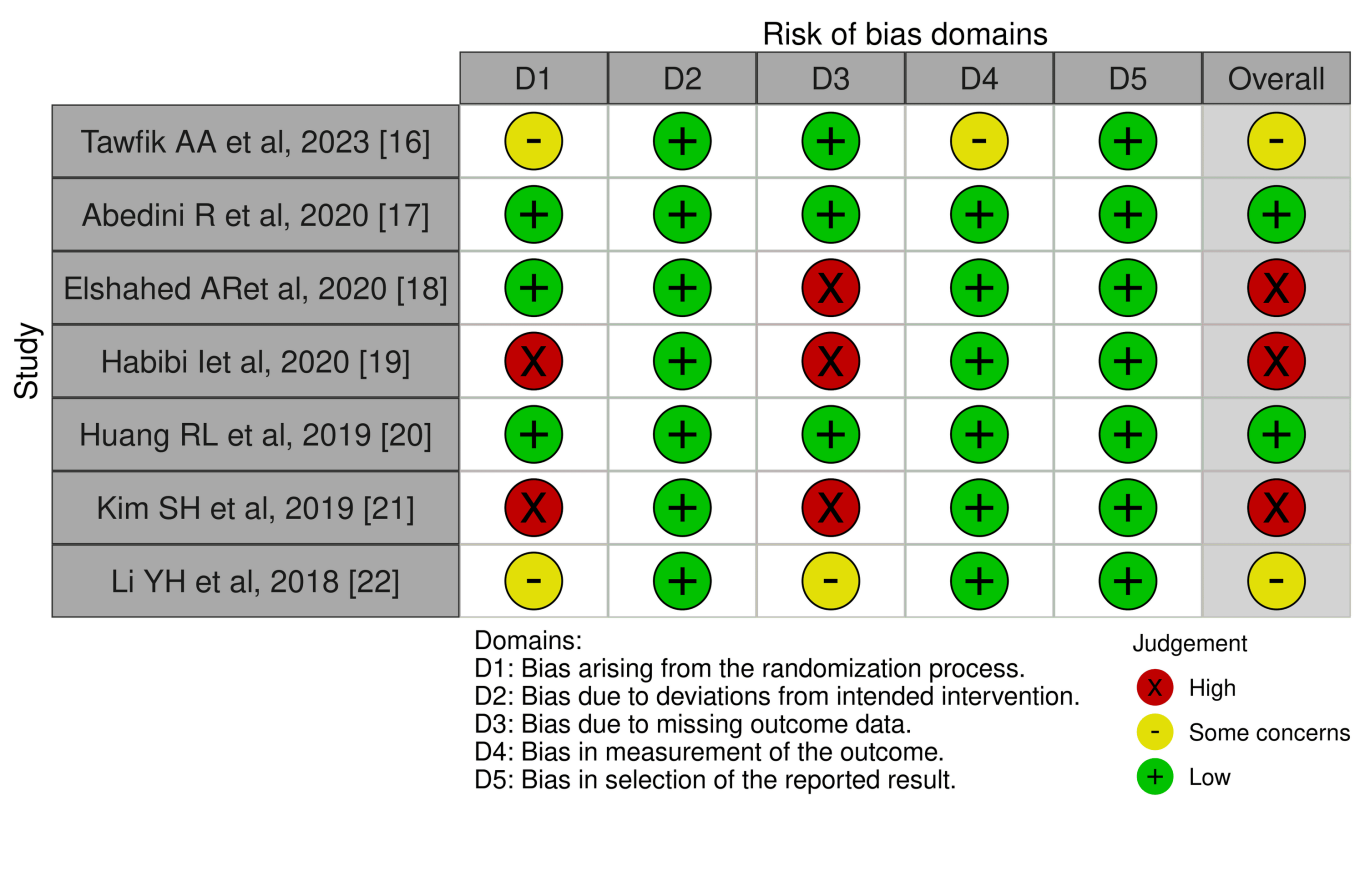
Traffic plot risk of bias assessment. Source: Tawfik et al. [16], Abedini et al. [17], Elshahed et al. [18], Habibi et al. [19], Huang et al. [20], Kim et al. [21], Li et al. [22].

**Figure 9:**
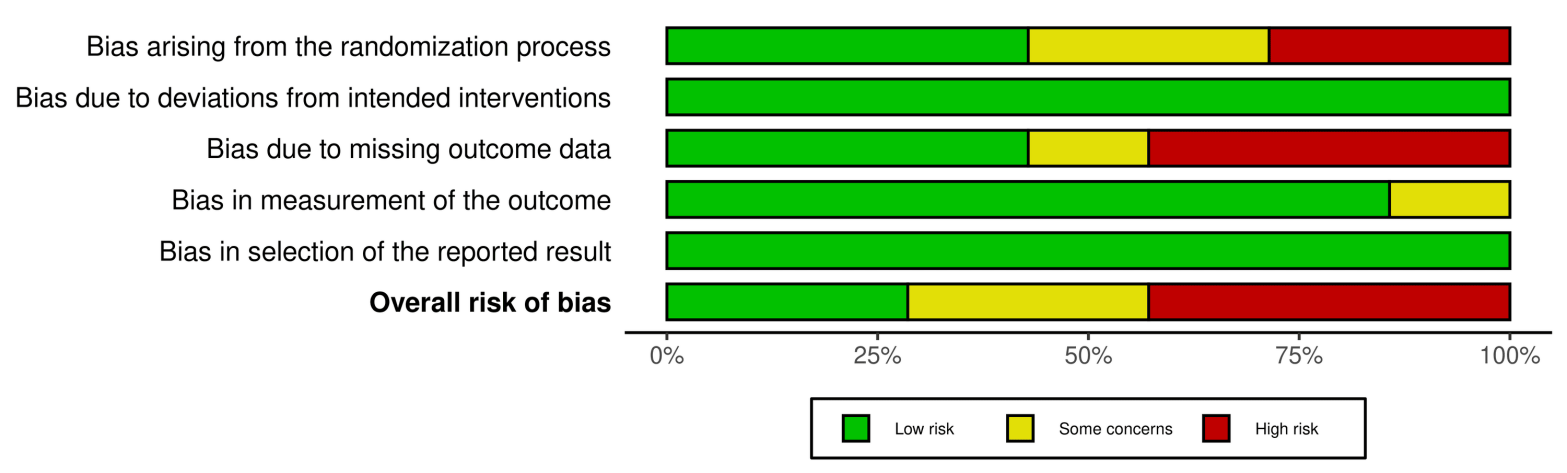
Risk of bias summary. Note: This image is the author's own creation.

Risk of bias assessment: The risk of bias assessment revealed that two studies conducted by Abedini et al. (2020) and Huang et al. (2019) showed low overall risk [13,16]. Two studies, Tawfik et al. (2023) and Li et al. (2018), raised concerns due to bias in randomization (D1), outcome measurement (D3), and outcome measurement (D4). Elshahed et al. (2020) [14], Habibi et al. (2020) [15], and Kim et al. (2019) [17], Figures 8, 9 show the risk of bias assessment of the included studies.

Discussion

This review provides an assessment of the efficacy of BTA in the treatment of hypertrophic scars. The analysis indicates several areas where BTA has potential benefits and areas where its effects are obvious. Our review focused on assessing the VSS as well as the individual parameters of the scale, which may have the highest contribution to the findings. The findings align with existing literature while also contributing new insights into the distinctions of BTA's therapeutic role.

VSS Scores and BTA

This review shows that there was a significant reduction in VSS scores associated with BTA treatment. The pooled mean difference of -1.69 (95% CI: -2.98, -0.40, P = 0.01) highlights that BTA is effective in enhancing the overall scar quality, as indicated by a reduction in VSS scores. This improvement in VSS scores, which assess parameters such as vascularity, height, pliability, and pigmentation, indicates the impact of BTA on scar characteristics. This result is consistent with the findings of Samarth et al. (2022) [23], who reported that BTA effectively reduces scar severity when used as an adjunctive treatment for both atrophic and hypertrophic scars. Additionally, Wang et al. (2022) [24] confirmed the safety and efficacy of BTA in improving the appearance of facial scars.

Scars Thickness and BTA

Regarding scar thickness, the effect of BTA appears to be less pronounced. The meta-analysis showed no statistically significant difference between BTA and placebo in terms of a pooled effect size of -0.03 (95% CI: -0.39, 0.32, P = 0.85). This suggests that while BTA may contribute to scar improvement in other dimensions, its impact on reducing scar thickness is limited. The findings revealed that the effect on scar thickness may vary depending on specific patient characteristics or scar types.

Other Parameters Including Scar Vascularity, Pliability, and Pigmentation

Furthermore, scar vascularity was another critical factor in scar assessment, and the studies included in this review provided mixed results regarding the efficacy of BTA in this parameter. The pooled mean difference indicated that BTA does not significantly affect scar vascularity compared to placebo. For example, Elshahed et al. (2020) reported a mean difference of -0.29 (95% CI: -0.68, 0.10), favoring BTA [18], while Huang et al. (2019) found an advantage for the placebo, with a mean difference of 0.05 (95% CI: -0.01, 0.11) [20]. This difference emphasizes the need for more standardized approaches to assess vascularity outcomes. It also shows that BTA's effect on this parameter may be dependent on several factors, including population and case factors. This was also confirmed by Yang et al. (2021) who revealed how differences in patient populations and treatment protocols affect outcomes in scar treatment, which may complicate the assessment and evaluation of BTA's efficacy [25]. 

Moreover, our analysis indicated that BTA significantly improved scar pliability, which is a critical parameter of functional and aesthetic scar recovery. The pooled mean difference (P = 0.02) indicated a statistically significant improvement in pliability with BTA compared to placebo. This may be attributed to the pliability or flexibility of the scar tissue, which can affect both the appearance and the function of scar therapy. In addition, the results are consistent with the findings of Yen et al. (2022) [26], who indicated that BTA's could reduce muscle tension in the area of the scar and could contribute to improved pliability and scar aesthetics.

Finally, the review analysis showed no significant effect of BTA on pigmentation, with a significant pooled mean difference (P = 0.66). This finding limits the role of BTA in improving skin pigmentation, which is aligned with previous research that has shown a lack of evidence for BTA's effectiveness in this area. Yen et al. (2022) found similar results, suggesting that while BTA may offer benefits in other areas of scar treatment, it does not significantly impact pigmentation [26]. This lack of effect on pigmentation points to the need for alternative treatments or combination therapies to address this aspect of scar healing.

The variability in outcomes across different studies underscores the complexity of using BTA for scar treatment. Factors such as the type of scar, the patient's age, the scar location, and the specific treatment protocol all appear to influence the effectiveness of BTA. The high heterogeneity observed in this review highlights the need for more standardized and rigorous clinical trials to better understand the optimal use of BTA in scar management. Additionally, because findings show that BTA may be most effective when used as part of a multimodal approach to scar treatment, addressing the physical characteristics of the scar and functional outcomes, such as pliability and scar quality, is crucial.

Long-Term Impact and Mechanism of Action of BTA in Scar Reduction

The long-term impact of BTA on scar reduction, in terms of collagen modulation and fibroblast activity, was emphasized in the study by Habibi et al. (2019). The findings showed that BTA did not significantly reduce all parameters of the modified Vancouver Scar Scale (mVSS); however, it did affect scar height, pliability, and vascularity over a six-month follow-up period. That is why the authors stated that BTA’s mechanism of action includes inhibition of fibroblast proliferation and reduction of the amount of extracellular matrix, which is obligatory for the formation of hypertrophic scars and keloids. BTA may further lessen the expression of transforming growth factor-beta 1 (TGF-β1) in fibroblasts, which may also explain for long-term effectiveness of the proposed BTA in scar management [19].

Consideration Regarding Pediatric Populations

The safety and clinical implications of BTA use in pediatric populations were addressed in the study by Tawfik et al. (2023) [16], which is considered the first to focus exclusively on children with post-burn hypertrophic scars. The study reported no adverse effects during the treatment or follow-up periods, affirming that BTA as a safe and effective treatment option for this vulnerable population. The parents and caregivers highly appreciated the improvements in scar pliability, vascularity, and height, particularly in cases where scars restricted joint mobility. These findings underscore the potential of BTA as a first-line treatment in pediatric scar management, especially when early intervention is critical for preventing long-term functional and cosmetic impairments.

## Conclusions

Thus, the current review concludes that BTA could be used as an effective treatment for the components of hypertrophic scars and keloids, especially regarding their pliability and quality. However, as for functions such as the change in the height of the scar, vascularity, and pigmentation, further research is still called for. More future research should focus on refining the treatment regimens, and the mode of action of BTA to the scar tissue, besides establishing the actual consequences of BTA treatment. These findings are supportive of prior studies and thereby shed light on the necessity of BTA in the management of scars.
